# Development of a urogenital scoring system and evaluation of macroscopic parameters influencing the sexual cycle in slaughtered sows

**DOI:** 10.1186/s40813-025-00458-1

**Published:** 2025-09-30

**Authors:** Philipp T. Egli, Julia Adam, Gertraud Schüpbach-Regula, Alexander Grahofer

**Affiliations:** 1 Clinic for Swine, Department of Clinical Veterinary Science, Vetsuisse Faculty, University of Bern, Bern, Switzerland; 2 Graduate School for Cellular and Biomedical Sciences, University of Bern, Bern, Switzerland; 3 Veterinary Public Health Institute, Vetsuisse Faculty , University of Bern, Bern, Switzerland

**Keywords:** Slaughterhouse, Pig, Reproductive system, Uterus, Ovary, Urinary bladder, Mucosa, Inflammation

## Abstract

Evaluation of the urogenital tract in slaughtered sows is a valuable diagnostic tool for reproductive disorders in pig breeding farms.Therefore, the aim of the study was to evaluate macroscopic parameters and their relationship to the reproductive cycle of the urogenital tract in slaughtered sows.

The following parameters were assessed in 105 randomly selected sows after slaughter: Weight of the empty bladder and the reproductive system (from the external urethral orifice to the ovary, with and without the broad uterine ligament). Furthermore, length of vagina, cervix and the right and left uterine horn were measured. The status of the reproductive cycle was evaluated by scoring the ovarian activity and the mucosa of the uterus and the urinary bladder was assessed for inflammation (Yes/No).

The average weight of the uterus was 1376.0 ± 623.3 g, and the mean weight of the bladder was 245.3 ± 100.8 g. The lengths of the left and right uterine horns were 148.7 ± 55.1 cm and 143.5 ± 55.4 cm, respectively. An inflammation in 43.8% of all uteri was detected and in 30.5% of all bladders. In the linear multiple regression model, a significant influence of the oestrus (*p* = 0.019), dioestrus (*p* = < 0.01), and the uterine mucosa (*p* = < 0.01) on the uterus weight was detected. Furthermore, a significant influence of the dioestrus (*p* = < 0.01), the uterine mucosa (*p* = < 0.01), and the bladder mucosa (*p* = < 0.01) on the bladder weight was detected.

The study provides current data on the urogenital tract of slaughtered sows. Considering the reproductive cycle, the weight of the uterus and the urinary bladder can be used as an indicator of inflammatory processes in the genital tract. However, these parameters should be interpreted in the context of macroscopic findings and not as isolated indicators.

## Introduction

Reproductive disorders are among the most common problems in modern pig production [1] and often lead to the culling of breeding animals; this, in turn results in reduced production of the farm. Early culling reduces the lifetime performance of the sows and, as a consequence, increases the costs for the farm [2, 3]. In addition, the longevity of animals is an important indicator for animal health as well as welfare and sustainability in pig breeding.

It is estimated that 30–50% of sows are culled due to reproductive failure, with major reasons including prolonged weaning-to-oestrus intervals, repeated returns to oestrus, and low conception rates [2, 4, 5] Slaughterhouse findings in culled sows frequently reveal pathologies such as endometritis, cystic ovarian degeneration, adhesions, and uterine abnormalities like hydrometra [5, 6]. These disorders typically present on farm as lack of oestrus, return to oestrus, small litter sizes, abortions, or mummified fetuses [7].

Reproductive disorders in sow herds can arise from a variety of causes. In most cases, fertility problems are non-infectious in origin. Genetic reasons [8], housing conditions [9], environmental factors [10], or feeding [11] are among the key elements that can significantly impact the reproductive performance of sow herds. However, infectious causes of infertility have also been described and must be considered in the clinical work up of reproductive problems on farm [7]. Therefore, an analysis of the reproductive performance characteristics together with a thorough herd examination with further diagnostic measures is required to make a final diagnosis.

An important diagnostic tool in porcine herd health management is the evaluation and examination of slaughter organs of affected animals. While specific systematic scores for pigs have been developed and validated for several organs such as the lungs [12, 13], pleura [14], kidneys [15], tail [16, 17] or claws [18], no standardized scoring approach is currently available for the urogenital tract. These organ specific scoring systems are used to evaluate post-mortem lesions in a standardized and reproducible manner, allowing for objective comparisons between farms and over time. For example, lung and pleura scoring systems help to identify respiratory disease patterns, whereas claw and tail scores are used as indicators for welfare related issues such as locomotion problems or tail biting. Such systematic evaluations serve as valuable herd health management tools by providing epidemiological insights and enabling targeted interventions. In contrast, current anatomical and macroscopic data on female urinary and reproductive organs in sows with reproductive disorders are still lacking in the scientific literature. Therefore, the aim of this study was to develop a scoring system for the evaluation of the urogenital tract in sows after slaughter, in order to systematically assess macroscopic parameters and their relationship to the reproductive cycle.

## Materials and methods

### Slaughterhouse material

**Fig. 1:**
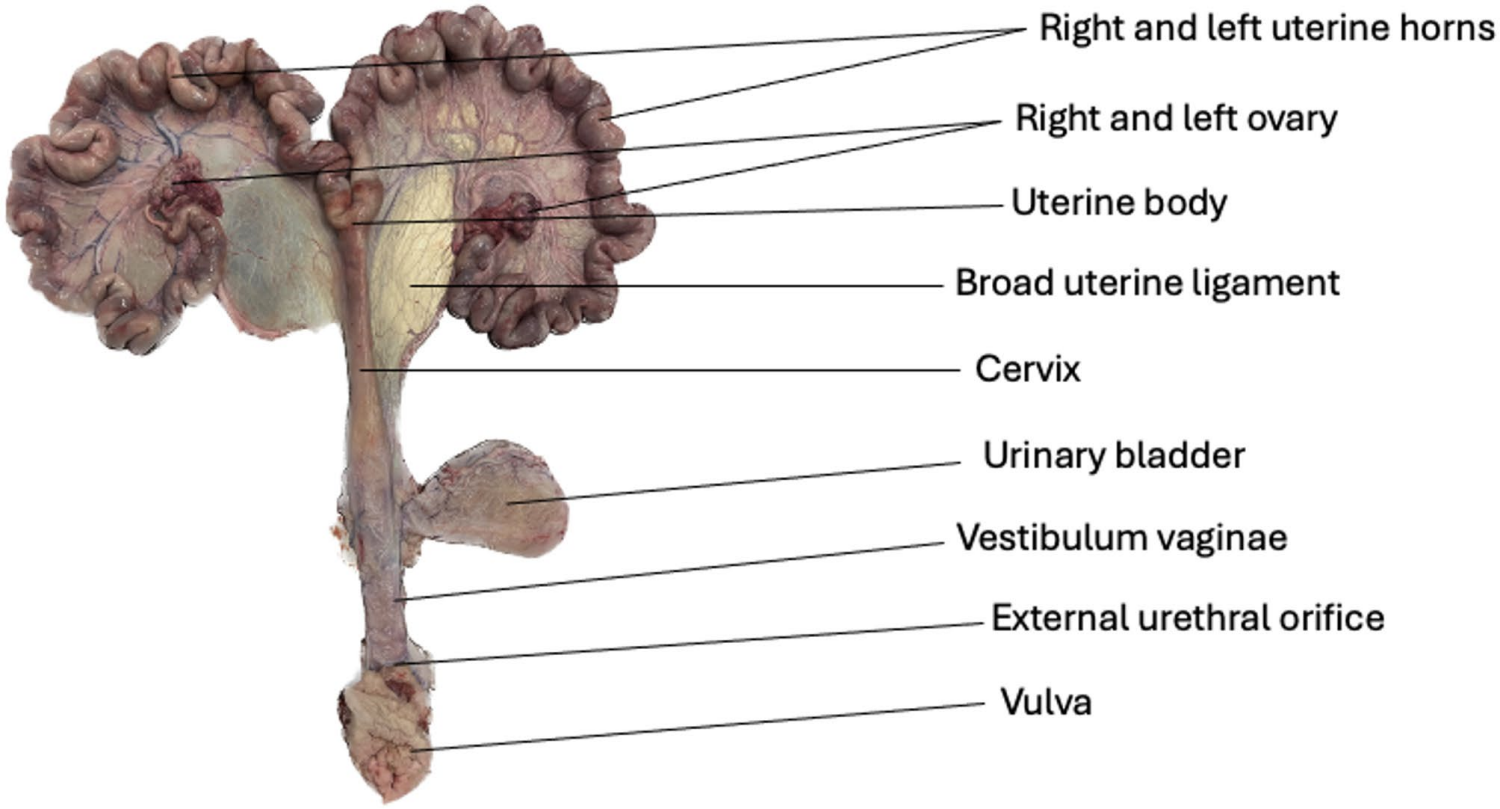
Urogenital tract of a culled sow. The reproductive system is shown from the external urethral orifice to the ovaries, including the vagina, cervix, uterine horns, and oviducts. The bifurcation of the uterus is indicated, corresponding anatomically to the point where the uterine horns diverge from the uterine body

Reproductive organs from 107 gilts and sows slaughtered in a Swiss abattoir were randomly collected on three different days in 2023. Since the animals were collected randomly at the slaughterhouse, no further details on breed, farm and culling reason were available. All sows were slaughtered in a standard sow-line of the slaughterhouse. The sows were electronically stunned, bled, and then brewed. Only animals suitable for human consumption were included in the study. The slaughter weight of each animal was recorded. The reproductive system (Fig. 1) with the urinary bladder were collected from a random sample of sows, and the individual identification was recorded for traceability. Afterward, the urogenital tracts were transported in a Leak-proof container and individually packed in a plastic bag and investigated in the lab (room temperature max 15 °C). Pregnant animals (*n* = 2) were excluded in the study. Therefore 105 sows and gilts were included in the study. The animals included in this study originated from typical commercial sow herds. The most common breed combination in Swiss pig production is a crossbreed between Swiss Landrace and Swiss Large White. After weaning individual confinement in insemination units is permitted only for a maximum of 10 days. Afterwards group housing of sows is mandatory by law In Switzerland. In addition, farrowing must take place in free-farrowing pens.

### Scoring system

The weight (g) of the empty urinary bladder was determined, the urinary bladder was opened, and the mucosa of the bladder was evaluated for macroscopic lesions.

**Fig. 2:**
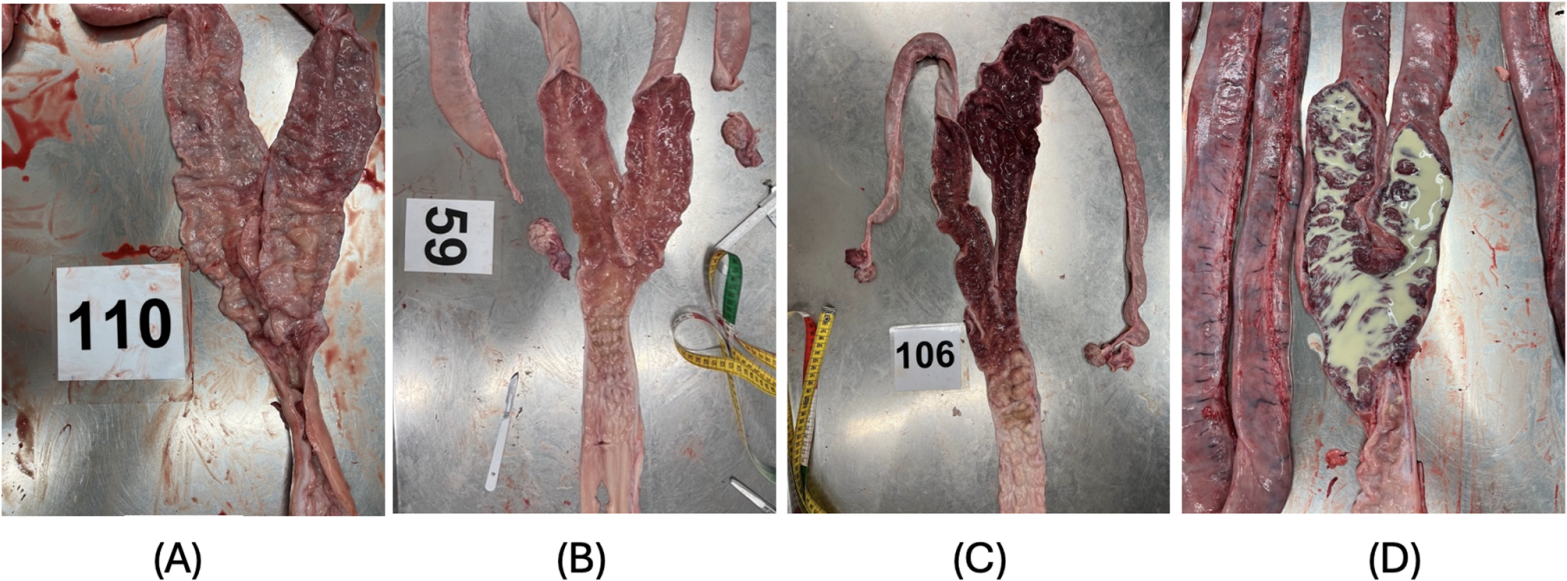
The mucous membrane of the uterus without macroscopic changes (**A**), (**B**) and with accumulation of fluid, severe redness (**C**) and pus (**D**)

**Fig. 3:**
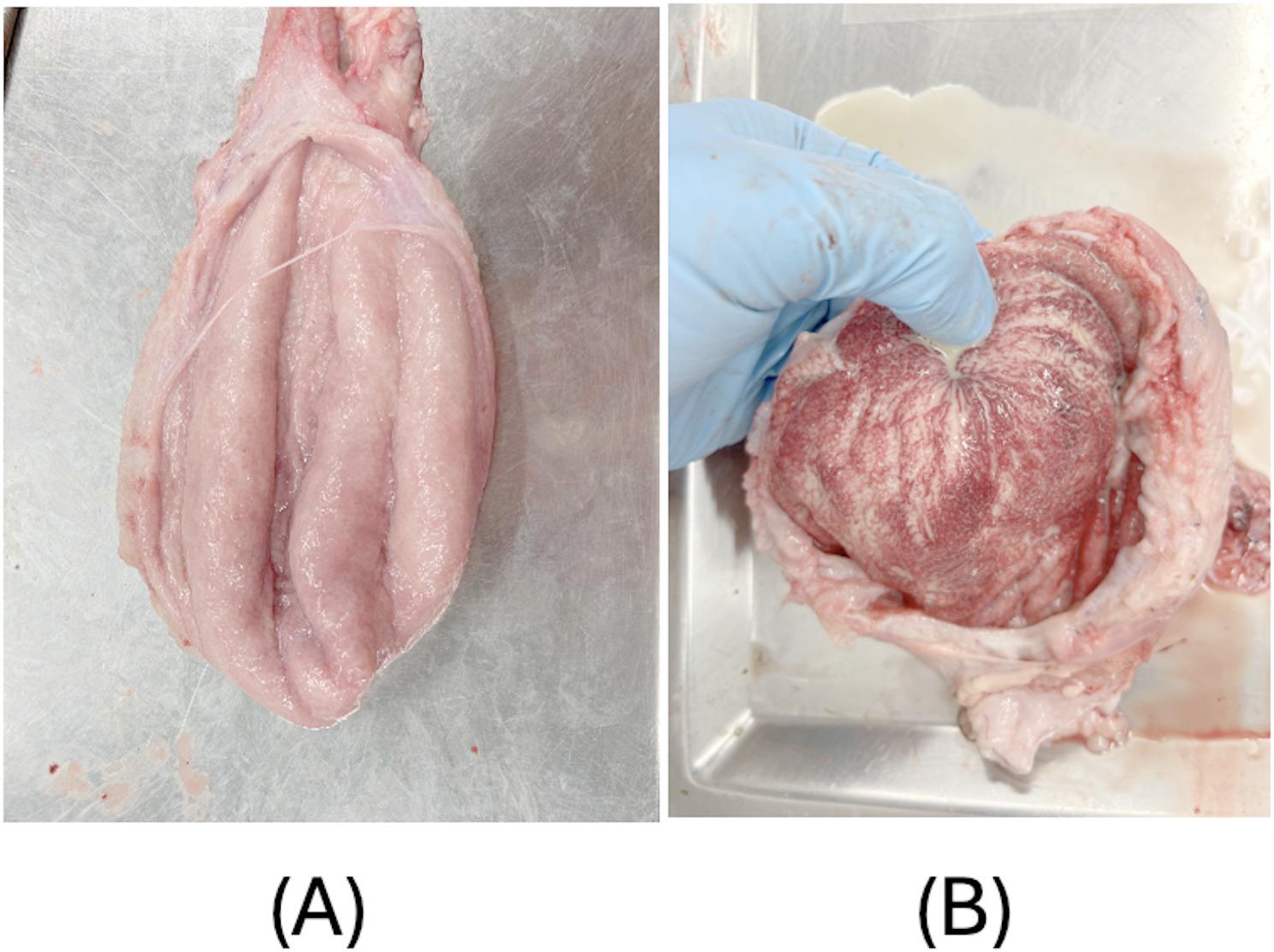
Scoring system for the mucous membrane of the urinary bladder. The figure (**A**) shows a mucous membrane without an inflammation, and the right figure (**B**) shows an inflammation

The vulva was removed and the weight of the reproductive system (from the external urethral orifice to the ovary, including the vagina, cervix, uterine horns and oviduct) was recorded with the broad uterine ligament. Afterwards, the broad uterine ligament was removed and the length of the vagina (cm), cervix (cm) and the right and left uterine horns (cm) were measured. In addition, the diameter (cm) of the cervix (widest part) and of the right and left lower uterine horn (ten centimetres from the bifurcation of the uterus) were measured. The mucous membrane of the uterus (Fig. 2) and the urinary bladder (Fig. 3) was examined macroscopically using the following scoring (Table 1). For both the uterus and urinary bladder, a binary scoring system (inflammation: yes/no) was applied based on the presence of any visible pathological changes such as redness, fluid accumulation, or pus. This simplified approach was chosen to facilitate objective, reproducible evaluation under field conditions. Although inflammatory severity may vary, as illustrated in Fig. 2 (D), we aimed to minimize observer bias by avoiding multi-level categorization at this stage of scoring development. This approach was chosen to enhance reproducibility and reduce subjectivity, particularly in settings without access to laboratory diagnostics.

**Table 1:** Scoring system for the mucous membrane of the uterus and the urinary bladder

Scoring		Findings on the surface of the uterus respectively the urinary bladder
No Inflammation		No macroscopic changes
Inflammation		Accumulation of fluid, severe redness, pus

The reproductive status of the ovaries was evaluated with a modified scoring according to [19] (Table 2).

**Table 2:** Classification of the cycle based on the findings on the surface of the ovaries

Cycle classification		findings on the surface of the ovaries
Oestrus animals		Follicle (F) with 5–15 mm
Dioestrus animals		Corpora lutea (CL)
Anoestrus animals		Inactive ovaries without CL or F less than 5 mm in diameter
Ovarian cysts		Cyst-like formations larger than 15 mm

**Fig. 4:**
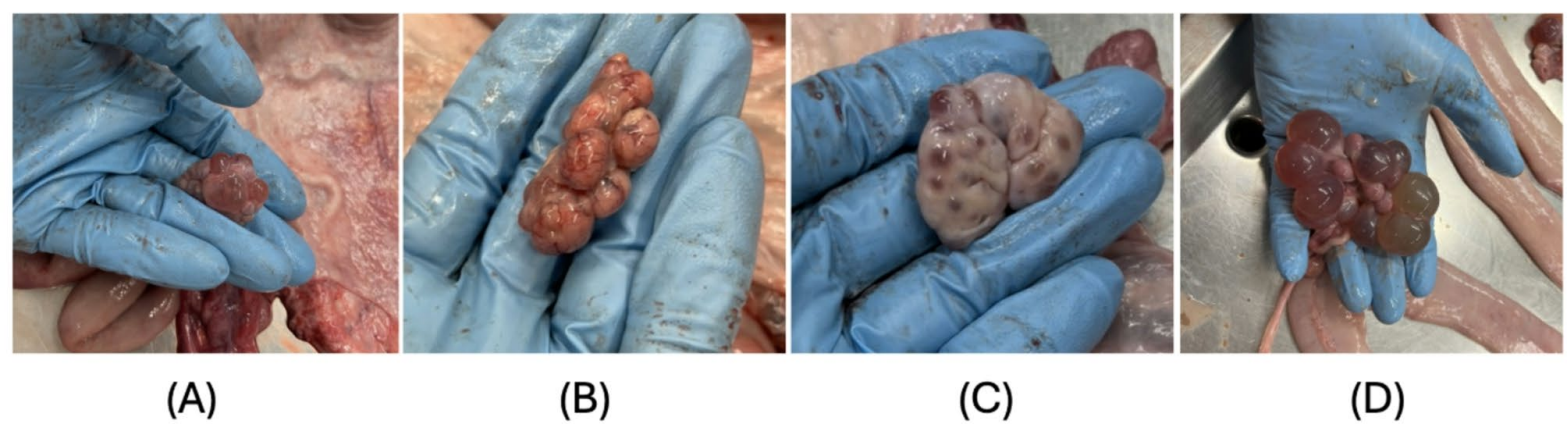
(**A**) Oestrus animals had follicles 5–15 mm in diameter. (**B**) Dioestrus animals had corpora lutea. (**C**) Anoestrus animals had inactive ovaries with neither corpora lutea nor follicles less than 5 mm in diameter. (**D**) Cyst-like formations larger than 15 mm

The functional bodies of the ovary were measured (Fig. 4): corpora lutea, follicles with a diameter larger than 5 mm, and any abnormalities (e.g. cysts). The animals were assigned to the reproductive cycle status according to their ovarian activity: Oestrus animals had follicles 5–15 mm in diameter and dioestrus animals had corpora lutea. Anoestrus animals had inactive ovaries with neither corpora lutea nor follicles of less than 5 mm in diameter. Cyst-like formations larger than 15 mm in diameter were recorded as ovarian cysts [19].

### Data management

All parameters were entered in a Microsoft Excel spreadsheet file (Version 16.83), sorted, and finally statistically analysed using the R Studio (Version 2023.06) [20]. A Shapiro-Wilk test was used to test each metric parameter for normal distribution. The parameters single ovarian cysts and multiple ovarian cysts were summarized as parameter cysts (1 = presence of cysts, 0 = No ovarian cysts) for further statistical analysis. For the parameter length of the uterine horn, the mean value from the left and right measurements was calculated for further consideration.

### Data analysis

In the descriptive analysis, the mean ± standard deviation, median and range were presented for metric data (slaughter weight, weight of the empty urinary, weight of the reproductive system, length of the vagina, length of the cervix, the length of the right and left uterine horns, diameter of the cervix, diameter of the right and left lower uterine horn, age, number of litters, number of live-born piglets all litters, number of live-born piglets last litter, number of stillborn piglets last litter, weaned piglets last litter).

For the categorical data (reproductive status of the sow, presence of cysts, abnormalities of the mucous membrane of the uterus, abnormalities of the mucous membrane of the urinary), the percentage of sows was presented in each case.

The continuous parameters were transferred to a scatter plot in R for a first assessment. A Welch Two Sample t-test was performed to compare the mean values of the lengths and the diameter of the uterine horns.

Furthermore, the linear relationship between weight of the reproductive system (g) and the average of the left and right uterine horn length using a Spearman’s correlation was calculated. This was conducted to check whether only the weight of the reproductive system could be used for further calculations. To classify the effect size of the correlation, an assessment was made according to a defined classification. The interpretation of the effect sizes according to [21] can be found in Table 3.

**Table 3:** Interpretation of the effect sizes

Effect size	Interpretation according to Cohen (1988)
|0.10|	Small effect
|0.30|	Medium effect
|0.50|	Large effect

Finally for weight of the empty urinary bladder and for the weight of the reproductive system respectively, multiple linear regression models were constructed. Reproductive status of the sow, presence of cysts, abnormalities of the mucous membrane of the uterus, abnormalities of the mucous membrane of the urinary were the independent variables. In R, the residuals of each parameter were checked (normal distribution, homoscedasticity). In addition, the correlation between the independent variables was checked to ensure that there was no multicollinearity. For the linear models, a log transformation for the parameter weight of the reproductive system and the weight of the empty urinary was performed to achieve normality of residuals.

## Results

### General data

An overview of all continuous parameters of the study population is shown in Table 4. Due to the damages during the slaughtering process only the length of the vagina from 104 animals and the length of the vulva from 98 animals could be measured. The average weight of the uterus was 1376.0 ± 623.3 g, and the mean weight of the urinary bladder was 245.3 ± 100.8 g. The lengths of the left and right uterine horns were 148.7 ± 55.1 cm and 143.5 ± 55.4 cm, respectively.

### Analysis for continuous parameters of the urogenital tract

The relationship between the slaughter weight and the weight of the reproductive system, revealing a significant positive correlation (*r* = 0.263, *p* = < 0.05). No correlation was detected between length of the vulva and the length of the vagina (*r* = 0.061, *p* = 0.551), the length of the vulva and the length of the cervix (*r* = 0.117, *p* = 0.251) and the length of the vagina and the length of the cervix (*r* = -0.072, *p* = 0.467). The length of the cervix correlated significantly with the length of the uterine horn (*r* = 0.465, *p* = < 0.05). In addition, a positive correlation (*r* = 0.215, ρ = 0.02) between the length and diameter of the cervix was shown. However, there was no correlation between the length of the vulva (*r* = -0.099, *p* = 0.334) and vagina (*r* = 0.115, *p* = 0.246) and the length of the uterine horn.

**Fig. 5:**
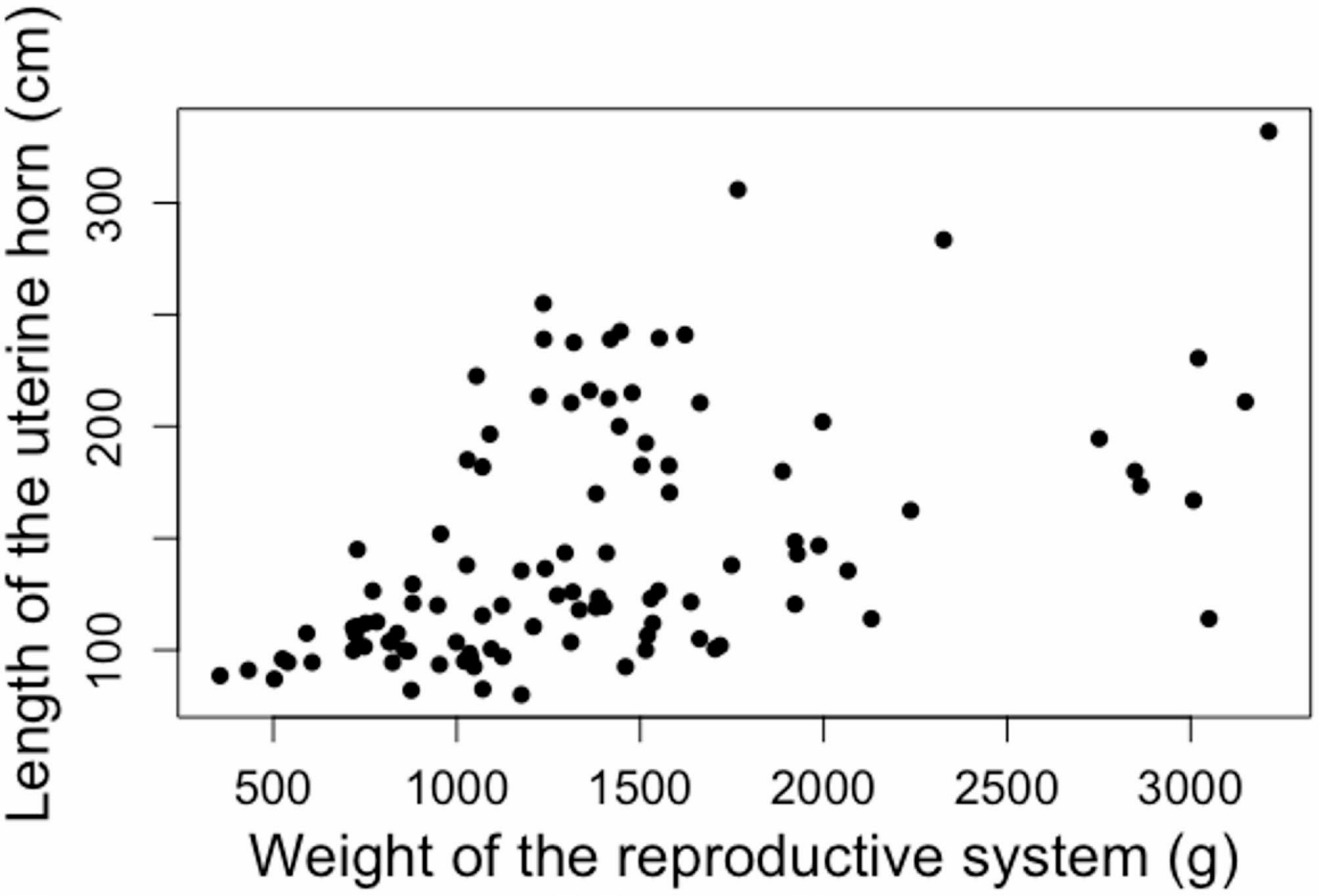
Relationship between reproductive system weight and uterine horn length

Furthermore, a moderate positive correlation between the weight of the reproductive system and the length of the uterine horn (*r* = 0.5574, *p* = < 0.05) was detected (Fig. 5).

The results of the Welch Two Sample t-test indicated that there was no significant difference between the mean lengths of the left (148.7 cm) and right uterine horns (143.5 cm) (t (208) = 0.677, *p* = 0.49), as well as, for the mean diameter of the left (2.8 cm) and right (2.8 cm) uterine horns (t (208) = -0.036, *p* = 0.971).

**Table 4:** Overview of the descriptive analysis for continuous parameters for all reproductive organs

Parameter	*n*	Mean ± SD	Median	Range
Slaughter weight (kg)	105	162.8 ± 31.0	164.3	93.5–245.5
Weight of the reproductive system (g)	105	1376.0 ± 623.3	1311.0	355.0–3213.0
Weight of the urinary bladder (g)	105	245.3 ± 100.8	226.0	87.0–847.0
Length of the vulva (cm)	98	8.9 ± 1.1	8.9	5.9–11.3
Length of the vagina (cm)	104	13.8 ± 3.3	13.5	5.0–28.0
Length of the cervix (cm)	105	24.6 ± 4.8	25.0	13.0–38.0
Diameter of the cervix (cm)	105	3.5 ± 0.7	3.5	2.1–5.3
Length of the left uterine horn (cm)	105	148.7 ± 55.1	128.0	78.0–329.0
Length of the right uterine horn (cm)	105	143.5 ± 55.4	120.0	78.0–335.0
Total length of the left and right uterine horn (cm)	105	292.2 ± 109.7	247.0	160.0–664.0
Diameter of the left uterine horn (cm)	105	2.8 ± 0.8	2.6	1.9–5.6
Diameter of the right uterine horn (cm)	105	2.8 ± 0.8	2.7	1.7–5.1

### Analysis for the binominal parameters of the urogenital tract

A summary of all binominal parameters of the study population is shown in Table 5.

**Table 5:** Overview of descriptive analysis for the binominal parameters for all reproductive organs

Parameter	*n*	%
Presence of paraovarian cysts	38	36.19
Presence of single ovarian cysts	5	4.76
Presence of multiple ovarian cysts	6	5.71
No ovarian cysts	63	60.00
Oestrus	12	11.43
Dioestrus	40	38.10
Anoestrus	53	50.48
Inflammation of reproductive system	46	43.81
Inflammation of urinary bladder	32	30.45

### Regression analysis of urogenital tract

**Fig. 6:**
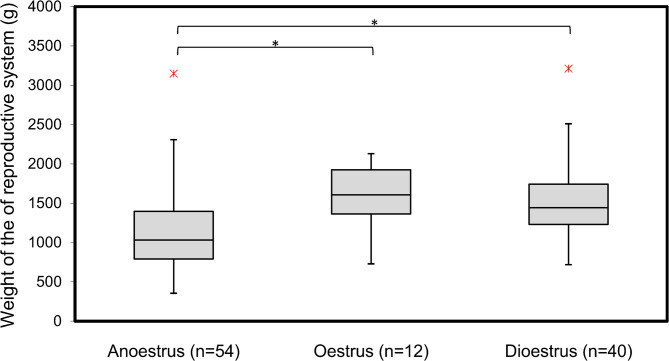
The relationship between the reproductive cycle and the weight of the of reproductive system

A regression analysis with the weight of the of reproductive system as the dependent variable and the parameters dioestrus, oestrus, inflammation urinary bladder, inflammation reproductive system and cysts as independent variables was conducted. The model showed that the included parameters were significantly associated with the weight of the of reproductive system (*r* = 0.282 *p* = < 0.001). The detailed results of the regression analysis are presented in Table 6. Dioestrus (*p* = < 0.001) oestrus (*p* = 0.003) and the Inflammation reproductive system (*p* < 0.001) had a significant influence on for the weight of the reproductive system. The relationship between the reproductive cycle and the weight of the of reproductive system is presented in Fig. 6.

**Table 6:** Results of the multivariable regression analysis for the weight of the of reproductive system

	β	95% CL		*p*	
		LL	UL		
Constant	6.823	6.697	6.950	< 0.001 *	
Dioestrus	0.320	0.159	0.483	< 0.001 *	
Oestrus	0.371	0.126	0.615	0.003 *	
Inflammation bladder	0.055	-0.109	0.219	0.508	
Inflammation reproductive system	0.274	0.118	0.429	< 0.001 *	
Cysts	0.062	-0.187	0.310	0.622	

R-square: 0.282; p-Wert: <0.001 *

Constant: Weight of the of reproductive system, Β: Regression coefficient,

CI: Confidence interval, LL: Lower limit, UL: Upper limit, p: *P*-value, * Level of significant < 0.05

**Fig. 7:**
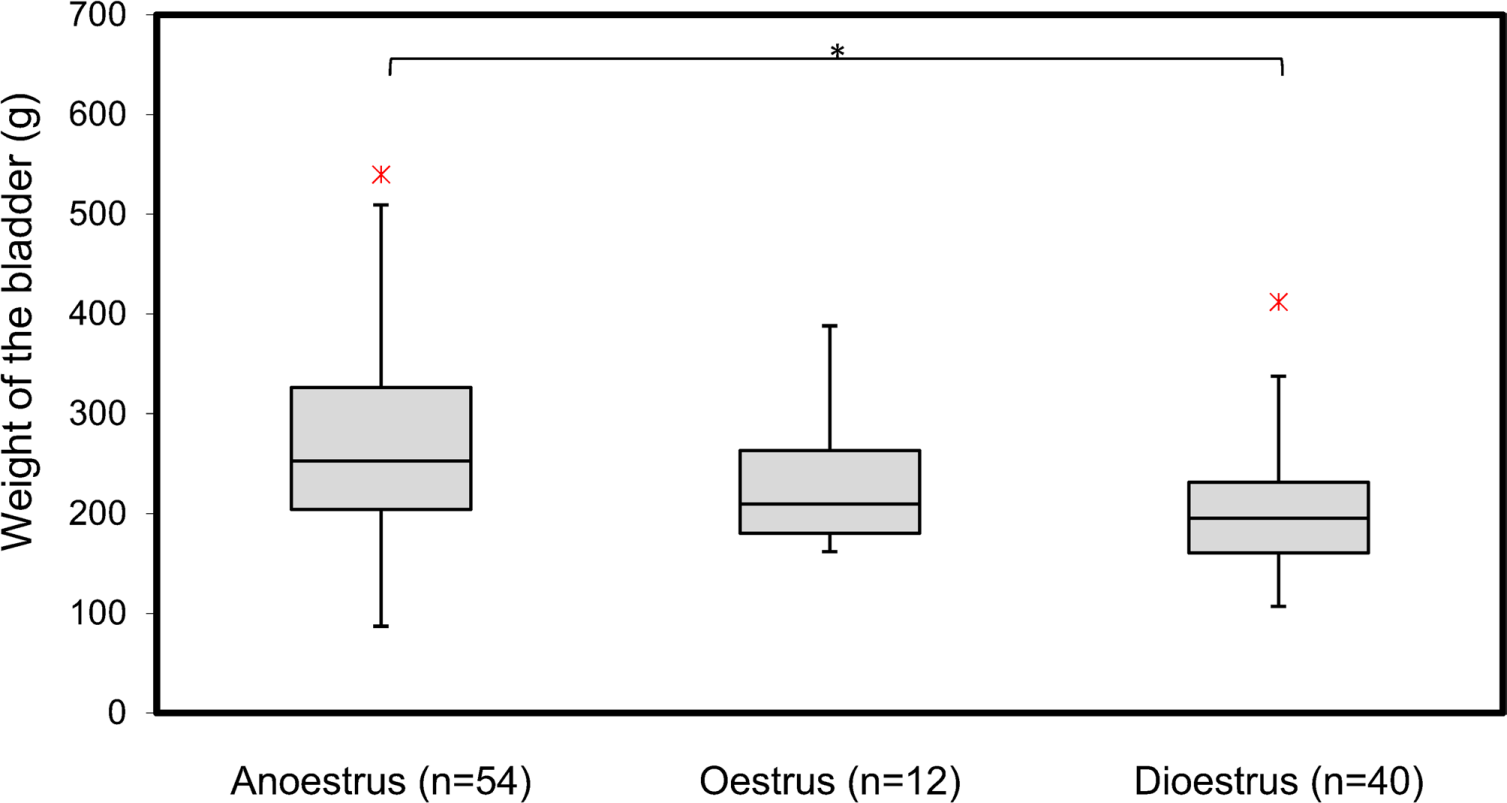
The relationship between the reproductive cycle and the weight of the bladder

A regression analysis was also done for the weight of the urinary bladder. The model showed that the included parameters were significantly associated with the weight of the of reproductive system (*r* = 0.296 *p* = < 0.001). The detailed results of the regression analysis are presented in Table 7. Dioestrus (*p* = < 0.001), the inflammation of the urinary bladder (*p* = < 0.001) and the Inflammation reproductive system (*p* = 0.032) had a significant influence on the weight of the urinary bladder. The relationship between the reproductive cycle and the weight of the urinary bladder is presented in Fig. 7.

**Table 7:** Results of the multivariable regression analysis for the weight of the urinary bladder

	β	95% CL		*p*	
		LL	UL		
Constant	5.404	5.317	5.531	< 0.001 *	
Dioestrus	-0.288	-0.409	-0.152	< 0.001 *	
Oestrus	-0.050	-0.239	0.145	0.611	
Inflammation urinary bladder	0.277	0.145	0.402	< 0.001 *	
Inflammation reproductive system	0.134	0.023	0.265	0.032 *	
Cysts	0.042	-0.172	0.068	0.671	

R-square: 0.296; p-Wert: <0.001 *

Constante: Weight of the of bladder, Β: Regression coefficient, CI: Confidence interval, LL: Lower limit, UL: Upper limit, p: *P*-value, * Level of significant < 0.05

## Discussion

This study evaluated the reproductive organs from 105 post-pubertal female pigs slaughtered in a Swiss abattoir and proposes a systematic evaluation protocol for the urogenital tract. Whereas findings of the urogenital tract of slaughtered sows have been described in previous studies, this to the authors’ knowledge is the first study conducted with sows from free farrowing and group housing systems.

The reproductive tract with the urinary bladder were collected from sows culled for non-verified reasons at various time points in the slaughterhouse. Since proper randomization of the study population was not achieved, selection bias was considered in the analysis. Furthermore, a limitation of the present study is that the sample may not be fully representative of live sows in production herds, as sows included in the study were likely culled, potentially due to reproductive failures or health issues. This selection bias could have contributed to the higher observed prevalence of disorders in the reproductive organs. However, the evaluation of the urogenital tract was conducted based on both qualitative and quantitative criteria. To minimize observer bias, all parameters were exclusively assessed by the principal investigator of the study. Although the sample size was limited to 105 animals, it allowed for detailed and standardized assessment of all specimens. The observed statistical associations support the relevance of the findings and encourage further research with larger sample size. Furthermore, no information on the animals’ age, breed, or reproductive history was available, which limits the interpretation of individual variability. These factors may have influenced the measured outcomes and should be addressed in future studies with more comprehensive animal-level data.

In the data set of this study, the average weight of the reproductive system was 1376.0 ± 623.3 g. In comparison with the literature, the average weight of the reproductive system was higher than in other studies [19, 22, 23]. However, it should be considered that two of the former studies [19, 22] were conducted over 25 years ago, while the study by [23] is more recent. The more recent findings by [24] report very similar values to those observed in the present study, supporting the notion that the reproductive tract of modern sows has increased in size. During this time period, genetic lines have evolved significantly, leading to improved reproductive performance. Especially, the number of total born have increased from 11 to 14 piglets up to 18–20 piglets nowadays [25] Literature indicates that the length of the uterus is associated with litter size [26], suggesting that the uterine capacity of hyperprolific sows has increased. This likely contributes to the higher weight of the reproductive organs observed today compared to the past. In future, it will be important to have a standardized procedure so that the data can be easily compared with each other.

In the present study, weight of the reproductive system was influenced by the reproductive status of the sow or gilt and the presence of inflammation of the reproductive system. The weight of the reproductive system typically increases during oestrus [27]. These changes in uterine weight are driven by hormonal changes, especially the rise in oestrogen levels during the oestrus phase [28, 29]. Oestrogen promotes blood flow to the uterus, leading to vascular engorgement [30]. During anoestrus, the hormonal changes characteristic of oestrus is absent [29]. Without the elevated oestrogen levels and the accompanying vascularisation and thickening of the uterine lining, the uterus weighs less [29]. It has also been shown that the weight of the reproductive organs is higher during dioestrus than during anoestrus. The progesterone level during dioestrus can increase uterine weight compared to anoestrus [31, 32].

The influence of different stages of the sexual cycle on the weight of reproductive organs in culled sows has been demonstrated in other studies [19, 23]. Additionally, the weight of the uterus can increase due to inflammatory processes. Inflammation leads to swelling and fluid accumulation, increasing the uterine weight [33]. Over time, the presence of infection can result in the formation of abscesses or other pathological changes that further contribute to the increased weight.

The mean weight of the bladder was 245.3 ± 100.8 g. This findings are in line with a recent study [34] which showed a bladder weight of 263 ± 89 g. Approximately one third (30.45%) f the sows sampled in the study were shown an inflammation of the bladder. There are hardly any studies on the macroscopic examination of the bladder. Similar prevalence (41% mld inflammation, 23% itense inflammation) of an inflammation of the bladder has been reported in a previous study by [34], which assessed mucous membrane of the urinary bladder in a similar way. In addition, the analysis revealed a significant positive effect of bladder inflammation and a significant positive effect of uterine inflammation on the weight of the bladder. This finding is consistent with the expected outcome, as inflammation typically leads to swelling and increased tissue mass [7, 35] and thereby increasing the weight of the bladder. A study on cystitis in sows and found significant associations between bladder pathology and increased bladder weight [34]. Specifically, bladders containing pus weighed 55 g more compared to bladders without these conditions. Additionally, intense redness in the bladders was associated with an 83 g increase in weight compared to bladders without redness. There was no difference between bladders without redness and those with mild redness. This shows, when evaluating bladder mucosa macroscopically, it is important to differentiate between mild and intense redness.

Inflammation of the reproductive system in sows is a significant concern in swine production, as it can impact fertility and overall reproductive performance. The results of this study indicate that 43.81% of the examined sows exhibited inflammation of the uterus, a notably high prevalence compared to findings in other studies. The binary scoring system proposed in this study represents a practical and easily applicable approach under field and slaughterhouse conditions. It relies on visible macroscopic changes such as redness, fluid accumulation, or purulent exudate to classify the mucosal surface of the uterus and urinary bladder as either inflamed or not inflamed. This simplified classification enhances reproducibility and reduces subjectivity, especially in settings where histopathological or microbiological confirmation is not available. Future studies should include comparisons with histological or bacteriological findings and assess inter-observer agreement to improve the diagnostic value and field applicability of this method.

For instance [6], reported endometritis in 14% of sows, while [22] observed a prevalence of 27%. However [5], documented a similarly high prevalence, with 52% of sows showing mild to severe inflammation. It is important to highlight that these previous studies [5, 6, 22] utilized a combination of macroscopic, bacteriological, and histopathological examinations, which may account for differences in reported prevalence rates.

The elevated percentage of uterine inflammation observed in this study remains unexplained. However, it may be influenced by differences in sampling procedure and in management and hygiene practices across various farms or regions.

The linear regression analysis provided information on the factors that influence the weight of the bladder. The presence of the dioestrus phase was associated with a significant decrease in bladder weight. In contrast, the oestrus phase did not show a significant impact on bladder weight. The interpretation of these results is difficult because there are no similar studies on the influence of the reproductive status on the bladder weight. However, it would be possible, that similar to the weight of the reproductive system, hormonal changes during the dioestrus phase can lead to decreased fluid retention or other physiological changes that result in a lighter bladder.

Ovarian activity is crucial for the reproductive performance in sows. It is well described that ovarian cysts as well as ovarian inactivity can result in infertility, cause irregular and prolonged oestrus cycles, and decrease conception rates [36].

A total of 50.48% of all animals were in anoestrus, which is considerably higher than the 22.9% reported by [19]. This elevated percentage may be attributed to the fact that most sows are slaughtered following the suckling period, a phase during which ovarian activity is typically low, leading to a higher prevalence of inactive ovaries [37].

In this study, approximately 10% of the culled sows exhibited single or multiple ovarian cysts. This is consistent with the literature, which reports a wide variation in the prevalence of ovarian cysts in sows, ranging from 2.4 to 15.95% [5, 19, 36, 38].

Interestingly, the dataset from this study revealed that 36.2% of the sows had paraovarian cysts, a value considerably higher than reported in other studies ranging from 4.0 to 22.9% [5, 19, 39]. To date, the impact of paraovarian cysts on sow reproductive performance remains unclear, as the number and size of these cysts can vary [40, 41]. However, it is speculated that interference with the oviduct may contribute to reproductive failure [41].

## Conclusion

The present study demonstrated current anatomical and macroscopic data on the reproductive tract of sows. The results show that the described procedure is a valuable diagnostic tool to evaluate macroscopic parameters and their relationship to the reproductive cycle of the urogenital tract in slaughtered sows. This study suggests that the weight of the uterus and urinary bladder may reflect inflammatory processes in the genital tract, especially when considered in relation to reproductive cycle stage. However, due to physiological variation among animals, these parameters should be interpreted in combination with macroscopic findings and not used as stand-alone indicators.

## Data Availability

No datasets were generated or analysed during the current study.
